# Effective Safety Message Dissemination with Vehicle Trajectory Predictions in V2X Networks

**DOI:** 10.3390/s22072686

**Published:** 2022-03-31

**Authors:** Hantao Li, Feng Liu, Zhongliang Zhao, Mostafa Karimzadeh

**Affiliations:** 1 School of Electronic and Information Engineering, Beihang University, Beijing 100190, China; lihantaobuaa@126.com (H.L.); liuf@buaa.edu.cn (F.L.); 2 Sensonic, 4780 Scharding, Austria; mos.karimzadeh@gmail.com

**Keywords:** vehicle trajectory density prediction, congestion prediction, effective transport system, safety data dissemination

## Abstract

Exploring data connection information from vehicle-to-vehicle (V2V) and vehicle-to-infrastructure (V2I) communications using advanced machine learning approaches, an intelligent transportation system (ITS) can provide better safety services to mitigate the risk of road accidents and improve traffic efficiency. In this work, we propose an end-edge-cloud architecture to deploy machine learning-driven approaches at network edges to predict vehicles’ future trajectories, which is further utilized to provide an effective safety message dissemination scheme. With our approach, the traffic safety message will only be disseminated to relevant vehicles that are predicted to pass by accident areas, which can significantly reduce the network data transmission overhead and avoid unnecessary interference. Depending on the vehicle connectivity, our system adaptively chooses vehicle-to-vehicle (V2V) or vehicle-to-infrastructure (V2I) communications to disseminate safety messages. We evaluate the system by using a real-world VANET mobility dataset, and experimental results show that our system outperforms other mechanisms without considering any predicted vehicle trajectory density information.

## 1. Introduction

Intelligent transportation systems (ITS) refer to systems that fuse information processing, wireless communication and sensor technologies to vehicles and transportation infrastructure to provide real-time information for road users and transportation system operators to make better decisions. The main goals of ITS are to improve traffic safety and efficiency via intelligent vehicle-to-infrastructure (V2I) or vehicle-to-vehicle (V2V) communications.

The initial motivation behind the development of V2X communications was to develop a road safety application to provide efficient early warning information and assistance to road users in order to prevent road accidents and disseminate accident information to relevant road users. From this perspective, knowing the future trajectories of the vehicles plays an important role in designing the safety message dissemination schemes.

Many solutions have been proposed to estimate the vehicle’s trajectory and density [1,2,3,4,5], which can be generally divided into two categories: infrastructure-based and infrastructure-less. The infrastructure-based approach requires some additional devices on the road networks, such as roadside magnetic loop detectors, surveillance cameras, wireless vehicle sensors, pressure pads, roadside radar and infrared counters, and so forth. Instead, the infrastructure-less approach requires only information obtained from the vehicle networks, such as vehicle to vehicle or vehicle to base station connection information or traffic flow information. Compared to infrastructure-based solutions, infrastructure-less solutions are more robust and reliable. Predicting the future trajectories of vehicles is important to support various types of ITS applications (e.g., improve communication capabilities and reduce vehicle congestion), but only a few efforts have been made to study how the estimated vehicle’s trajectory can be used to enhance wireless communications or improve traffic efficiency. In addition, few studies have focused on the combination of V2V and V2I communications to enhance the system performance or to overcome infrastructure failures without compromising system reliability.

In this work, we tackle the two problems of vehicle trajectory prediction and efficient safety message dissemination by integrating V2V and V2I communications simultaneously. We present a system that is able to predict the vehicle trajectory and explore the trajectory prediction information to improve the safety message dissemination mechanism in an efficient way. Our proposed vehicle trajectory prediction algorithm is based on a hybrid Markov chain approach, which is able to switch between the first or second order Markov chain according to the mobility trace quality. With the predicted future locations, road accident information will only be disseminated to vehicles that are predicted to pass by the accident areas. The message dissemination mechanism will select direct V2V communication to transmit safety messages if the end-to-end vehicle connection is available. In case the network is sparse and interested vehicles can not be reached via V2V communications, the system will automatically switch to V2I mode to inform the relevant vehicles to guarantee the timely delivery of the safety messages. The main contributions of this work can be summarized as follows:

- We propose an end-edge-cloud architecture to deploy the machine learning-driven vehicle trajectory application on the network edge. The vehicle trajectory prediction scheme is based on a hybrid Markov chain approach with an inverted index model.
- We present a hybrid early warning system together with a multi-hop data dissemination protocol for VANETs based on vehicle trajectory prediction to deliver alert messages to relevant vehicles in both sparse and dense scenarios. Our system can adaptively select either the V2V or V2I connections to disseminate the warning message based on the vehicle connectivity information to guarantee the timely delivery of the warning message.
- We evaluate our trajectory predictor, hybrid early warning system, and multi-hop data dissemination protocol using a rich vehicle dataset collected from a real-world VANET testbed. Through comprehensive experiments, we obtain consistently satisfactory results.

This paper is organized as follows. Section 2 discusses existing work on vehicle trajectory prediction in VANETs and its application in VANETs. Section 3 explains the proposed system architecture, the vehicle trajectory prediction algorithm, as well as the trajectory prediction-based safety message dissemination scheme. Section 4 describes the experiments and evaluation methodology. Section 5 analyzes the experimental results. Finally, Section 6 presents the concluding remarks.

## 2. Related Work

This section presents the related work of a vehicle’s trajectory and density prediction, and discusses their applications in intelligent transportation systems with their advantages and drawbacks.

### 2.1. Trajectory and Density Prediction

Accurate density estimation of On-Board Units (OBUs) in urban environments is important for various applications in VANETs: safety-related applications are expected to reduce the risk and severity of accidents, and warn a driver whenever a collision at an intersection is probable; efficiency-related applications aim at managing the traffic flow on roads and monitoring vehicles’ movements; and comfort-related applications aim to provide entertainment facilities and up-to-date contextual information for passengers by means of Internet access while traveling in urban areas. These applications could be more efficient if they become aware of the density of OBUs at any given time and place [6]. Knowing the accurate density of OBUs in a vehicular communication environment is essential for these applications because a vehicle should be able to adapt its behavior according to a vehicle’s density anytime and anywhere due to the high mobility and dynamics of VANETs [1]. Therefore, several research efforts have been made to study the predictability of vehicle density in trajectories as discussed in the following. The first studies to predict the density of vehicles used a Kalman filter to extract traffic density by monitoring camera images [2] or estimating the traffic density by utilizing cumulative acoustic signals collected from a roadside-installed microphone in the road segments [7]. However, applying these approaches is very costly, and in real-world urban areas, only a small fraction of the road segments are covered by sensors. For those road segments without sensors, those methods may be no longer applicable. In addition, the damage of an installed sensor is another shortcoming that should be considered. To deliver better prediction results, some studies explore models that rely on the vehicle’s movement patterns. A novel model to forecast traffic flow that depends on the acceleration and velocity of vehicles is presented in [8]. Online learning weighted support-vector regression (OLWSVR) [3] integrates a regression model with a weighted learning method. Bastani et al. [9] studied urban traffic estimation by analyzing the number of vehicles located in the transmission range of road-side units (RSUs). These density estimation approaches are not robust enough for accurate vehicle density estimation in urban areas, since they rely on vehicles’ movement information (e.g., acceleration variation, velocity or direction of movement), which includes many complicated interactions over the roads and involved crowds. Due to the non-linear and stochastic nature of traffic, some proposals have used deep learning methods [10], such as the deep belief network [11] and stacked autoencoder (SAE) [12]. Most of them perform traffic prediction for highways, where traffic flow is relatively stable. In addition to the aforementioned methods, the type-2 FL model [13], infinite-mixture model [14] and dynamic traffic assignment [15] were also used to estimate traffic flow in city areas. However, the main drawback of these models is their inability to estimate the specific time of predicted density in trajectories, since these models only work on spatial granularity (*location of congestion*), which means that the algorithm outputs only the number of vehicles at each specific segment of a trajectory without any timing information. To overcome this shortcoming, we propose a model that improves prior methods with the ability to predict the density of vehicles in trajectories by estimating the time of congestion. Moreover, the proposed technique does not demand costly actions as opposed to existing deep learning models.

### 2.2. Intelligent Traffic Management

Several solutions have been proposed to deal with mobility issues including traffic congestion [16,17,18,19], unexpected traffic incidents [20,21,22] and vehicular traffic re-routing [23,24,25,26]. Services such as INRIX [27] provide real-time traffic information, which might support drivers to choose their routes. In turn, Google Maps and Waze are Vehicular Navigation Systems (VNSs) that can recommend faster routes based on a global traffic view whenever a route plan is requested [28,29]. On the other hand, vehicular re-routing based solutions focus on recommending periodically faster routes to vehicles to improve the overall traffic efficiency.

Those approaches try to improve traffic efficiency by recommending faster routes based on current traffic conditions on the roads, but their performance potentially decreases during unexpected traffic incidents, since they do not implement any pro-active mechanism to deal with such specific issues. Unexpected traffic incidents, such as vehicle crashes, can dramatically decrease the mobility efficiency if not properly handled [29,30]. In this scenario, vehicles affected by such unexpected incidents, for example, vehicles that will pass by the accident location, need to be notified as soon as possible to take some actions such as change the route or delay their departure to minimize the effects introduced by this unexpected event.

To tackle this issue, most proposals rely either on multi-hop data dissemination approaches [16,20,24] or on infrastructure-based approaches [21]. Multi-hop data dissemination approaches consider only vehicle-to-vehicle (V2V) communications, thus assuming a fully connected network to send notification warnings to target vehicles. In infrastructure-based approaches, RSUs are responsible for delivering the notifications to the set of target vehicles, which need to be covered by RSUs. However, for most cities, it is far from reality to consider having a fully connected vehicular network all day long in such a dynamic environment as well as full RSU coverage. Moreover, the delivery of early warnings could be a very difficult task [28]. For instance, in sparse scenarios, data dissemination-based approaches need to replicate warning notifications periodically throughout the whole duration of an unexpected incident to ensure that they will deliver the warning to all target vehicles. This approach potentially overloads the network with unnecessary transmissions and decreases the overall system effectiveness. In some cases, vehicles might receive the warning too late, not allowing them to take alternative routes to improve their mobility. In principle, infrastructure-based approaches cannot work if the unexpected incident occurs in some area not covered by RSUs.

Wang et al. [21] proposed NRR, an adaptive next road rerouting system for unexpected urban traffic congestion avoidance. NRR saves the cost of obtaining a global traffic view by relying on local information available at RSUs (which are assumed to be deployed at each intersection) to select the best next road for each vehicle. The idea is to avoid the road that contains an unexpected incident rather than computing a whole new route. The local information is built based on the vehicles’ report. In order to detect unexpected congestion, NRR relies on a central server responsible for sending a notification to the RSU closest to the congestion. Thus, the RSU broadcasts such a notification to all vehicles within its coverage, consequently enabling them to verify whether their routes go toward some road that will potentially become congested. The RSU uses the latest obtained traffic information to compute the next road, so that vehicles can avoid an unexpected incident based on their local traffic view. However, having an RSU deployed at each intersection of the city for delivering the service efficiently is not a realistic assumption due to geographical and economic issues.

In our previous work, we proposed ICARUS [20], an intelligent system to improve traffic conditions based on an alerting and rerouting system. ICARUS is aware of both the current traffic conditions and unexpected traffic incidents. Therefore, when it detects any traffic congestion or an unexpected traffic incident, it creates a warning message and spreads it through the network (based on a predefined area of interest) to warn vehicles about the incident. ICARUS employs a delay-based data dissemination protocol, which addresses the broadcast storm problem by relying on a broadcast suppression mechanism based on the *sweet spot* concept. In addition, to ensure that all vehicles receive the warning, ICARUS periodically rebroadcasts it. Finally, to avoid congestion, whenever a vehicle receives a warning message, it verifies if it will pass through the congested area or the area with the unexpected traffic incident and requests a new route to a central server that possesses a global traffic view. Yet ICARUS uses only V2V communications to deliver the warning for the vehicles that will pass by the accident, thus delivering an early warning in partitioned scenarios (e.g., scenarios with network partitions) might not be possible, and in some cases it can also decrease the system’s efficiency due to late delivery.

We consider the aforementioned issues found in the literature such as limited performance when dealing with unexpected traffic incidents [16,23,24,25,27], the deployment of RSUs at each intersection of the city [21], limited coverage and latency in partitioned scenarios [20], and do not consider future trajectories and density to improve their services [16,20,21,23,24,25,27,28]. We propose an intelligent safety message dissemination mechanism that offloads network traffic whenever it is possible (e.g., multi-hop data dissemination) and delivers early warning messages in partitioned scenarios, through infrastructure-based data forwarding considering the RSUs deployed at the scenario and the vehicle’s trajectory. To do so, we employ an efficient vehicle’s trajectory and road density predictor to enable the system to know in advance the network scenario (e.g., connected or partitioned), and the set of vehicles that need to receive the early warning about some unexpected traffic incident to greatly improve network utilization and system performance. Therefore, our system addresses the following challenges: (i) how to predict the vehicles’ trajectory and traffic density; (ii) how to decide when to use a multi-hop data dissemination approach or infrastructure-based solution to deliver early warning messages; (iii) how to identify the set of vehicles that need to receive the early warning notification; and (iv) how to perform efficient multi-hop data dissemination.

## 3. Safety Message Dissemination with Vehicle Trajectory Prediction

### 3.1. System Overview

In this section we provide an overview of the proposed system, which is designed to deliver reliable traffic warning messages to relevant vehicles using V2V multi-hop data dissemination or infrastructure-based V2I forwarding whenever V2V connections are not available. The system includes a vehicle trajectory and density prediction mechanism, which estimates future locations and densities of vehicles such that only vehicles that are predicted to pass the accident areas will be informed about the en-route unexpected traffic incident.

**Figure 1:**
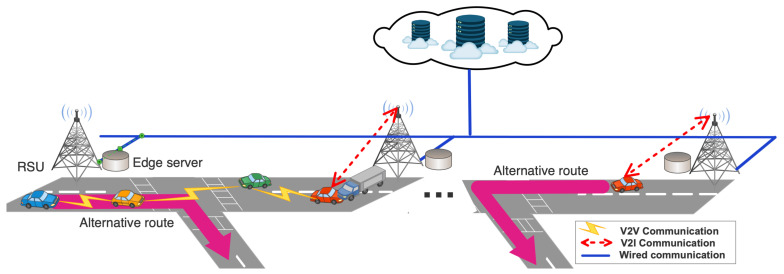
V2X Safety Message Dissemination System overview.

As shown in Figure 1, the system is based on a hybrid architecture composed of vehicles as OBUs, edge servers as RSUs, and centralized cloud servers. OBUs are responsible for detecting unexpected incidents, notifying nearby edge servers, and disseminating warning messages. RSUs, which are working as edge servers, are responsible for running vehicle trajectory and density predictions using pre-built models, and deciding whether to deliver the warning messages to relevant vehicles using V2V or V2I approaches. The cloud servers are responsible for managing global network information and performing complex data analyses, such as building machine learning models that will be used by edge servers to make real-time trajectory and density predictions.

Whenever an OBU detects an unexpected traffic incident (e.g., such as engine damage, air bag activation, hard breaking), it sends an unexpected traffic incident notification to the nearest RSU either using the cellular network or the IEEE 802.11p protocol. In this way, the trajectory and density prediction functions running in edge or cloud servers will be triggered such that the RSUs can identify vehicles that will pass the detected traffic incident area and should receive the early warning. Additionally, from the trajectory and density prediction the system can know vehicles’ future locations and consequently the connectivity among vehicles. This enables the system to transmit the early warning messages to relevant vehicles using either a V2V multi-hop data dissemination approach or a V2I solution when the V2V connectivity is not available.

On the one hand, for the vehicles that can receive the early warning through V2V communication, the RSU notifies the source vehicle (the one that sent the massage to the RSU) to start the multi-hop data dissemination using an efficient algorithm (described later) that selects the best relay vehicles towards the destination based on density predictions. On the other hand, for those vehicles that should receive the message (they are predicted to pass the accident areas) through the infrastructure, two cases can occur: (i) the target vehicle is within the coverage area of another RSU; and (ii) the target vehicle is not within the coverage area of any RSU. In the first case, the early warning is forwarded to that RSU and delivered to the vehicle. In the latter case, we rely on the mobility prediction to know which are the next RSUs that the target vehicle will pass. These are the RSUs that will receive the early notification to increase the delivery rates and as soon as the target vehicles are connected to them, they will be notified immediately. Finally, when the early warning message is delivered to the target vehicle, it can change its route using a re-routing algorithm to avoid that particular area (e.g., the unexpected incident location).

**Figure 2:**
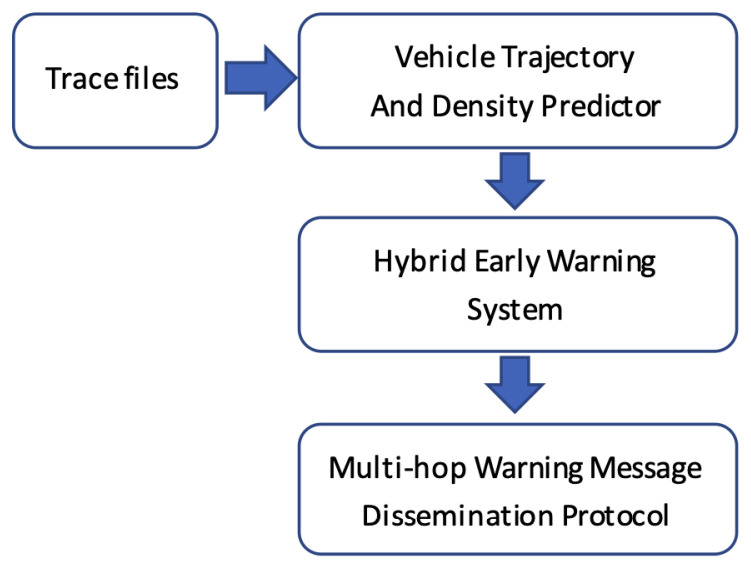
Flow chart of the proposed system.

Therefore, the proposed system includes three main components: (i) vehicle trajectory density prediction; (ii) a hybrid early warning system; and (iii) an intelligent multi-hop message dissemination protocol, as described in the next sections. Figure 2 shows the flow chart of the proposed system. It is worth noticing that the proposed system is built on top of the dataset collected from real-world VANET testbed deployed in Porto, Portugal [31,32].

### 3.2. Vehicle Trajectory and Density Prediction

**Figure 3:**
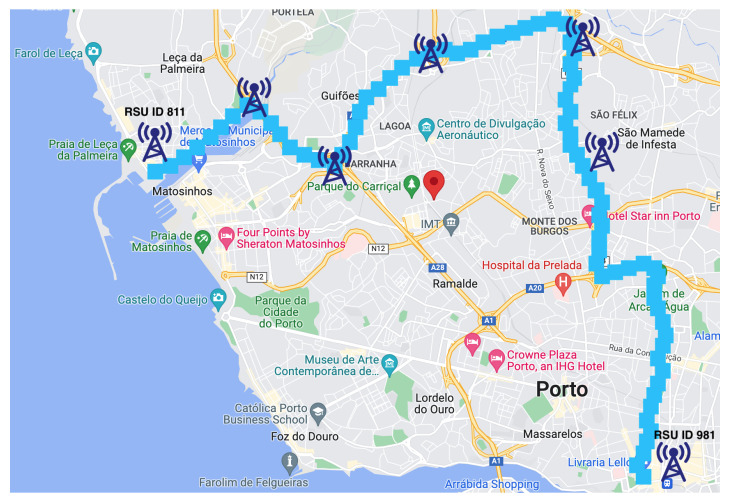
Partitioned trajectory between two locations for OBU-ID = 2007.

A vehicle’s trajectory is defined as the route from one location to another one in an urban area [33]. Vehicles can take multiple routes to move among different locations. To discover the trajectories, we explored a rich vehicle dataset collected from a real-world VANET testbed deployed in the city of Porto, Portugal [31,34]. The testbed consists of 600 OBUs and 120 RSUs. This dataset includes the actual OBU–RSU connectivity from October 2016 until August 2017, which enabled us to discover the vehicles’ trajectories. The set of discovered paths of each single vehicle among two RSU $ID_{i}$ and RSU $ID_{j}$ was recorded in $T_{i,j}$ = $\left[t_{1},t_{2},\ldots ,t_{n}\right]$. The raw data collected from the VANET testbed includes a large amount of information. However, for OBU’s trajectory extraction, we only needed the list of sequentially connected RSUs, global positioning system (GPS) coordinates of each connected RSU and the time stamps of the connections. Therefore, we had to generate a file with all the connected RSU IDs as well as timestamp information for every OBU. The data points were received at a sampling rate of 20 to 40 s, and the maximum distance between two sequential location points was 15 m. The first five fields indicated the timestamps of the connections, the sixth field indicated the connected RSU ID, and the last two fields indicated the *latitude* and *longitude* of the connected RSU. After generating the trace files for each OBU, the next step was to discover the trajectories. We used the Google Maps API Direction Service (https://cloud.google.com/maps-platform/ (accessed on 1 January 2022)) to discover all possible routes between two consecutive RSUs. By specifying a starting RSU (*connected RSU at time t*), a destination RSU (*connected RSU at time t+2*) and a way-point RSU (*connected RSU at time t+1*), the Direction Service can return the route using the way-point. By determining a way-point, the Direction Service can calculate a more accurate path between origin and destination. After discovering the routes, the next step was to partition the geographical space into grid cells, for which we used the Python Google S2 Geometry Library (http://s2geometry.io/ (accessed on 1 January 2022)). Each grid cell was a four-corner cell, which covers a specific region. Generated grid cells were accumulated into a set of Cell = {$c_{1}$, $c_{2}$, …, $c_{m}$}. Each observed path $t_{k}$ was partitioned into a sub-list of *l* visited grid cells. The resulting partitioned path can be shown as $t_{k}:\left\{c_{1},c_{2},\ldots ,c_{l}\right\}$. Given the *GPS* coordinates, the location of any moving object at a specific time can be mapped to a grid cell. In this work, we defined a grid cell with of a size of 300 m^2^. Each grid cell was identified by a 64-bit Cell ID. As shown in Figure 3, the discovered trajectory for the OBU ID 2007 among two locations (RSU ID 811 and 981) using way-points in the city center of Porto was partitioned by successive grid cells.

Collected mobility traces of vehicles during their movement could be used to explore some regularities in their travels in the city. This knowledge was used by a density predictor to forecast the number of vehicles that may take the same trajectories in the future. The two key aspects for density prediction in trajectories are: (i) predicting the vehicles’ future locations over time (connected RSU); and (ii) estimating the future trajectories between predicted RSUs for multiple vehicles during the same time interval (e.g., next 1 min). The collected mobility trace, which includes connected RSU IDs and timestamps for each vehicle, was used to train our mobility predictor to predict the next connected RSU. The implemented mobility predictor constantly chooses the first order or the second order Markov chain, based on the quality of mobility trace to forecast future locations of the vehicle [35,36]. The mobility predictor has a tunable time threshold, which enables the algorithm to determine the next connected RSU ID of each vehicle in scales of minutes or hours. After predicting the next movement of each vehicle, our trajectory predictor attempted to estimate the future trajectory that each vehicle was going to take.

**Figure 4:**
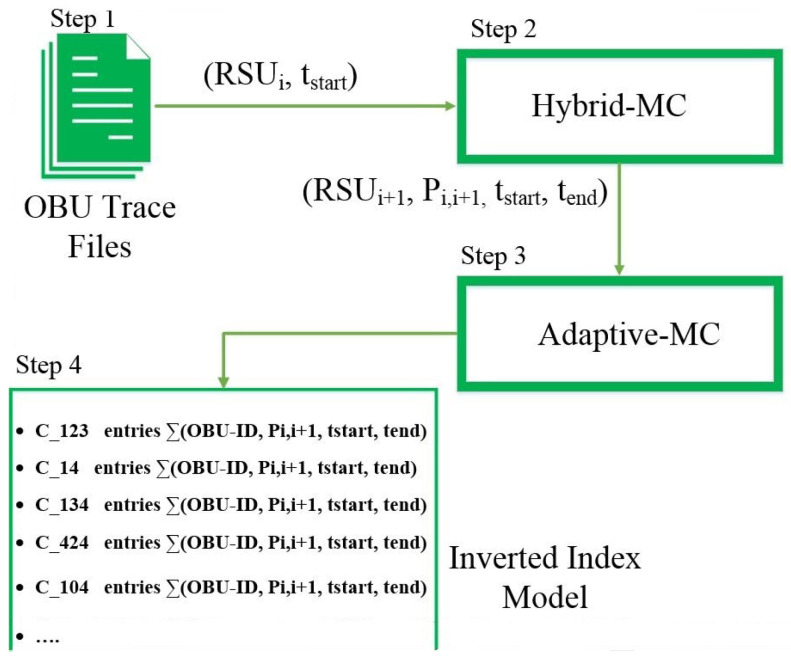
Vehicle trajectory density predictor.

As shown before, each trajectory was partitioned as a series of grid cells between two sequential RSUs. The trajectory predictor was introduced in our previous work [33], in which the sequence of visited grid cells by each moving object was the input of the model. The first order and the second order Markov based algorithms are available predictors. The proposed model can adapt its behavior continuously based on the availability of grid cells and the behavior in the city to maximize the prediction performance. By storing and aggregating the predicted trajectories for multiple vehicles, we could estimate the OBUs that will meet each other in a certain grid cell. To do so, we used an inverted index model to store the predicted trajectories of multiple vehicles during the same time interval. The model includes *n* rows, where *n* refers to the number of unique grid cells in all predicted trajectories for a specific time slot. Figure 4 depicts the proposed vehicle’s trajectory density predictor, which consists of three main units: hybrid Markov chain as the mobility predictor, adaptive Markov chain as the trajectory predictor, and the model, which is based on an inverted index model. The next four steps enabled us to estimate the density of vehicles along the trajectories.

(1) Input for our model: mobility trace of each single vehicle with the connected RSU IDs and connection time.
(2) Output of our model: after finishing the training step, the hybrid Markov chain generates a 4-item tuple (RSU $ID_{i+1}$, $P_{i,i+1}$, $t_{start}$, $t_{end}$), where RSU $ID_{i+1}$ is the predicted RSU that the OBU is going to connect, $P_{i,i+1}$ represents the transition probability to move from RSU $ID_{i}$ to RSU $ID_{i+1}$, $t_{start}$ and $t_{end}$ are the timestamps to indicate when the vehicle was connected to current RSU (RSU $ID_{i}$) and when the vehicle will arrive at the next RSU (RSU $ID_{i+1}$), respectively. The connection time to a future RSU is estimated by the hybrid Markov chain.
(3) Using the current RSU $ID_{i}$, which can be easily obtained from the trace for each vehicle and the estimated RSU $ID_{i+1}$ from Step 2, the trajectory between these two RSUs can be predicted using the adaptive Markov chain. The output of the algorithm is an ordered list of grid cells, which the OBU will pass through when moving from RSU $ID_{i}$ to RSU $ID_{i+1}$.
(4) As soon as the predicted trajectory among two RSUs is identified, the inverted index should be updated. To do so, first, each row in the inverted index model is indexed by each grid cell ID of the predicted trajectory. Then, the tuple (OBU ID, $P_{i,i+1}$, $t_{start}$, $t_{end}$) is stored in the corresponding row of the inverted index model. Expired tuples for each vehicle are deleted from the rows. Finally, by summing up the number of tuples stored at each row, we can estimate the number of vehicles that may pass through the respective grid cell from $t_{start}$ to $t_{end}$.
  The output of the proposed trajectory density predictor is stored in $TDp_{i,j}$ = {($c_{1}$, *P*-OBUs), ($c_{2}$, *P*-OBUs),…, ($c_{l}$, *P*-OBUs)}. The set includes two item tuples, where the $c_{l}$ ∈ {$c_{1}$, $c_{2}$, …, $c_{m}$} demonstrate grid cells on the predicted trajectory that is passing from RSU $ID_{i}$ to RSU $ID_{i+1}$, and *P*-OBUs store the OBU IDs that we estimated to meet each other on the specific grid cell of the predicted trajectory.

### 3.3. Effective Safety Message Dissemination

With the trajectory density prediction, the system knows in advance the future position of each OBU and OBU densities. This enables it to identify: (i) the communication scenario (e.g., connected or partitioned); (ii) the set of vehicles that will pass a traffic incident location whenever this occurs; (iii) the most suitable way to send the warning to each vehicle that needs to receive it, considering multi-hop data forwarding and infrastructure-based forwarding; and (iv) the set of the most suitable relay OBUs to perform multi-hop data forwarding.

Thus, whenever an OBU detects an accident, it notifies the closest RSU, such that the RSU can start the procedures to send the early warning to vehicles that need to be informed. Algorithm 1 describes the main procedures of the early warning mechanism. In this scenario, based on the current position of the OBU that has detected the incident, the system can extract its location (e.g., its current cell $c_{i}$) (Line 2). Therefore, to identify the communication scenario (e.g., connected or partitioned), the system needs to create an undirected graph G=(V,E) representing the communication network based on the trajectory density prediction $TD_{p}$ (Line 3), in which the set *V* represents the OBUs while the set *E* represents the communication links between them. In other words, considering the position of each OBU, the system can identify the set of OBUs that are connected together and can communicate to each other by means of multi-hop data forwarding. This identification is based on the communication range of each OBU and the distance among them (e.g., the distance between two OBUs is smaller than the their communication range). Therefore, to identify the set of vehicles that needs to receive the early warning $OBU_{Targets}$, the system predicts the trajectory $T_{i,j}$ of each OBU and checks if they will pass by the accident location (e.g., $c_{incident}\in T_{i,j}$) (Lines 5–8). With the set of target vehicles ($OBU_{Targets}$) available, the system can determine how to deliver the early warning to each target OBU. In this case, if there is a path in the communication graph from the source OBU to any target OBU, the system will forward the early warning using a V2V multi-hop data dissemination (Lines 11–13). Otherwise, the early warning is sent using the RSUs. Thus, the system needs to predict the set of RSUs that each target vehicle will pass by to forward the early warning to them to increase the delivery probability (Lines 15–17).
**Algorithm 1:** Hybrid early warning system.
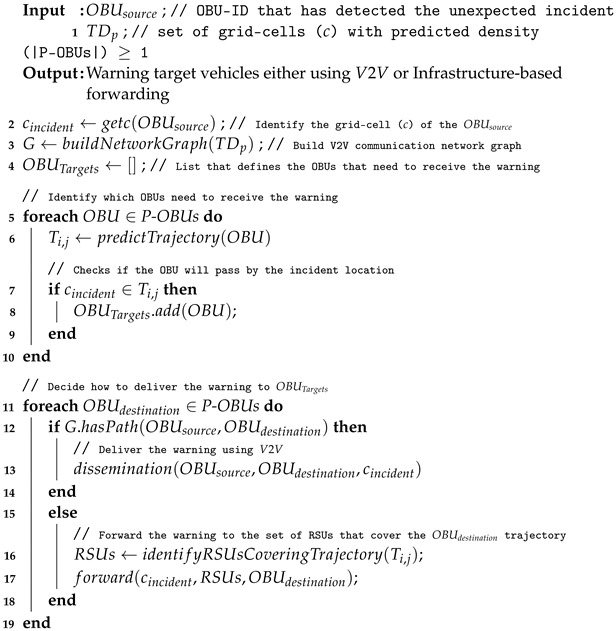


Algorithm 2 describes the efficient multi-hop data dissemination based on mobility and density predictions. Considering the origin $OBU_{source}$, the destination $OBU_{destination}$ and the communication graph *G*, the system is able to select the best path (e.g., the best set of relay OBUs) to deliver the early warning. To do so, it builds a sub-graph Links based on the k-Shortest Paths [37] from $OBU_{source}$ and $OBU_{destination}$ to identify the set of possible paths that the warning message can be sent using multi-hop forwarding (Line 3). In this way, the system selects the next OBU based on the set of neighbors in the Links sub-graph and the distance to the destination of each neighbor to determine the set of relay OBUs. Therefore, the next OBU will be the neighboring OBU that is closest to the destination. The set of relay OBUs is computed iteratively from $OBU_{source}$ towards $OBU_{destination}$ (Lines 6–9). The distance-based approach maximizes the progress of every forwarding operation and reduces the number of transmissions to guarantee the effective warning message delivery.
**Algorithm 2:** Data dissemination algorithm based on the trajectory and density prediction.
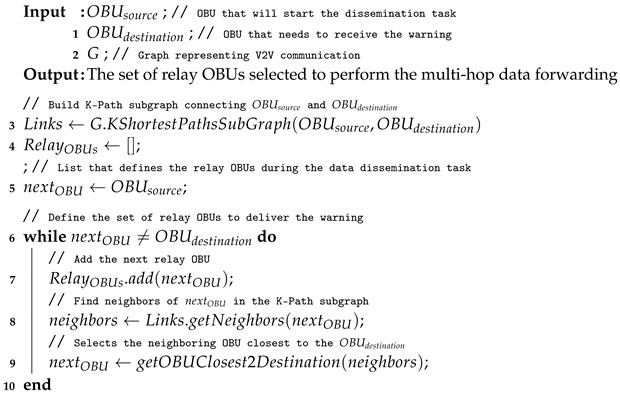


Finally, whenever a target vehicle receives an early warning, it can employ a re-routing algorithm to change its route and avoid the incident area to improve its mobility. A new route changing algorithm is out of the scope of this paper. Therefore, we have implemented the efficient route changing mechanism employed by our previous work [20]. This route changing approach considers the ongoing traffic conditions (e.g., based on the density of each road) and the location of the unexpected incident to compute an alternative route. In addition, to avoid the problem of creating another congestion spot by computing the same alternative route for many vehicles with the same origin and destination (limitation presented in deterministic re-routing approaches), it uses a probabilistic approach to select a route in a set of possible alternative routes.

## 4. Experiment Setup

In this section, we describe the experiment details and evaluation methodology to examine the performance of the proposed trajectory density predictor and its impacts on the safety message dissemination mechanism.

### 4.1. VANET Mobility Dataset

As described in Section 3.2, the proposed trajectory density predictor for this work integrates both mobility and trajectory predictors to estimate the number of vehicles that may meet each other in grid-cells. In order to train and also test our hybrid and adaptive Markov chains, we need mobility trace datasets. In this research, we used a real-world VANET testbed deployed in the city of Porto, Portugal [31,32]. This large-scale dataset consists of 600+ networked OBUs using IEEE 802.11p, WiFi or 4G as well as 120+ RSUs scattered along the city. The dataset includes different pieces of information, such as RSUs information (*RSU location, RSU ID, volume of the download/upload traffic via 4G, WiFi, DSRC, etc.*) and OBU information (*connected RSUs, OBU ID, timestamps, transmitted 4G/DSRC traffic, etc.*). However, for trajectory density prediction, only RSU IDs, GPS coordinates of connected RSUs and timestamps are required. To evaluate the prediction performance of our mobility predictor, we split the trace data as 70% for learning and 30% for testing. To train our adaptive Markov chain to estimate future trajectories for every single OBU, the 10-fold cross validation was performed.

### 4.2. Evaluation of Vehicle Trajectory Density Prediction

To evaluate the effectiveness of the proposed vehicle density predictor, we used the *accuracy* metric. This metric is commonly used as an evaluation measure in *information retrieval* [38], which indicates the relationship between the numbers of True Positive (TP), True Negative (TN), False Positive (FP), and False Negative (FN), which are defined in Equations (1)–(4), respectively.

(1) $$TP=c_{i}\in TDpi,j\wedge c_{i}\in TDri,j,$$

(2) $$TN=c_{i}\notin TDpi,j\wedge c_{i}\notin TDri,j,$$

(3) $$FP=c_{i}\in TDpi,j\wedge c_{i}\notin TDri,j,$$

(4) $$FN=c_{i}\notin TDpi,j\wedge c_{i}\in TDri,j,$$

where $TDp_{i,j}$, $TDr_{i,j}$ are the predicted density of vehicles in a trajectory by the density predictor and the real density, which are extracted directly from the dataset, according to the vehicles that take the partitioned trajectory to move from the location of RSU-$ID_{i}$ to the location of RSU-$ID_{j}$, respectively. We introduce the function *Accuracy($TDp_{i,j}$, $TDr_{i,j}$)* to quantify the prediction performance of the proposed algorithm, in (5).

(5) $$\begin{array}{c} Accuracy(TDp_{i,j},TDr_{i,j})=\\ \;\frac{TP(TDp_{i,j},TDr_{i,j})+TN(TDp_{i,j},TDr_{i,j})}{\left(TP(TDp_{i,j},TDr_{i,j})+FN(TDp_{i,j},TDr_{i,j})+FP(TDp_{i,j},TDr_{i,j})+TN(TDp_{i,j},TDr_{i,j})\right)}. \end{array}$$

### 4.3. Evaluation of Traffic Accident Message Dissemination

**Figure 5:**
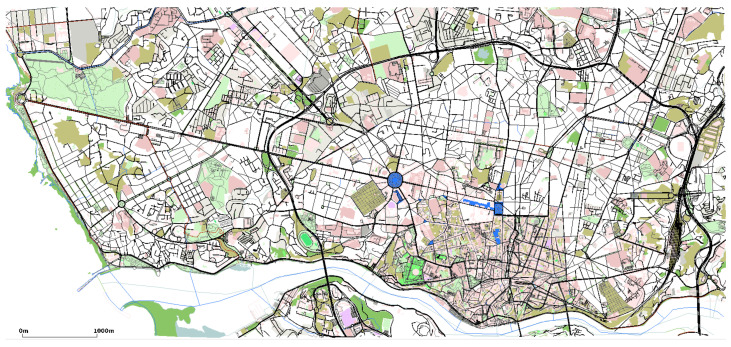
SUMO scenario of Porto map extracted using OSM.

**Figure 6:**
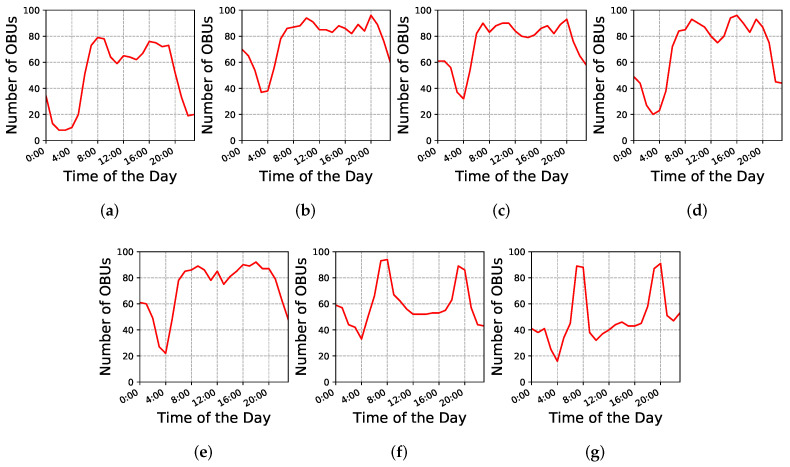
Scenario density along each day of trajectories extracted from the dataset.(**a**) Monday; (**b**) Tuesday; (**c**) Wednesday; (**d**) Thursday; (**e**) Friday; (**f**) Saturday; (**g**) Sunday.

To evaluate the proposed hybrid early warning system, first we need to produce a simulation platform based on the real-world VANET dataset. The simulation platform is composed of: (i) the mobility simulator SUMO [39], which provides the urban mobility and vehicular traffic management using a Traffic Command Interface (TraCI) framework; (ii) the network simulator OMNeT++ [40], which is a discrete event-based simulator that provides a set of tools for developing network applications; and (iii) the vehicular network framework Veins [41], which implements the IEEE 802.11p standard and extends SUMO and OMNeT++ simulators to offer a comprehensive suite of models for developing ITS and VANET application and protocols. To produce the same characteristics of the real-world VANET dataset, we extracted the Porto city map using the OpenStreetMap (OSM) tool and converted it into a SUMO scenario. Moreover, to produce the same traffic presented in the dataset we used the predicted trajectories, i.e., based on the centroid coordinates of each $c_{i}$ that composes the OBU trajectory we mapped it to the closest road present in the scenario. In this case, we were able to employ the proposed hybrid early warning system and evaluate its performance considering traffic and network based metrics. For the sake of clarity, Figure 5 and Figure 6 show the extracted SUMO scenario using OSM and its traffic density for one week, all day long, considering the trajectories exported from the dataset.

With the simulation platform, we analyzed the system’s performance by randomly introducing a set of 100 unexpected traffic incidents into the scenario considering different days and periods. To produce an unexpected traffic incident, for each day and period chosen, we randomly selected an OBU from the mobility trace. Thus, we randomly selected a grid-cell (e.g., $c_{i}$) from its trajectory in which the OBU will make an unexpected stop, thus the vehicle in the simulation stays stopped in that $c_{i}$ closing the entire road for one hour, and, after that, the road is released [42]. Therefore, as soon as the unexpected traffic incident occurs, we trigger the proposed hybrid early warning. As simulation parameters, we set the bitrate to 18 Mbit/s at the MAC layer and the transmission power to 2.2 mW, resulting in a communication range of approximately 300 m under a two-ray ground propagation model. Table 1 summarizes the main simulation parameters used in our assessment.

**Figure 7:**
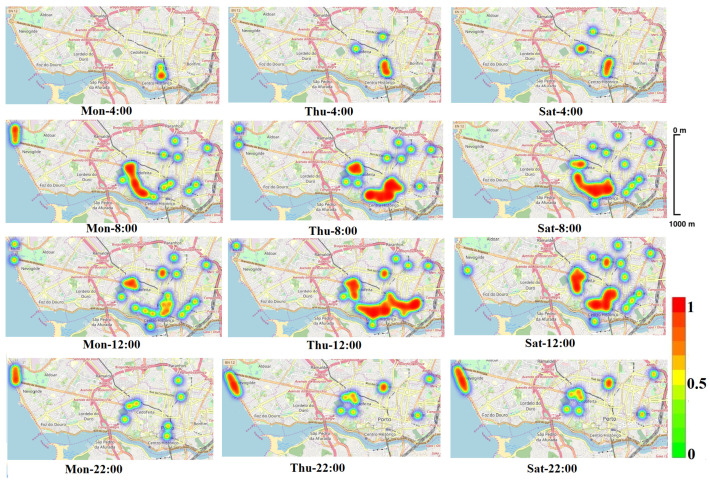
Trajectory density prediction at different times of different days.

In this dataset, we observed that there are potentially two communication scenarios for all weekdays, which can be broadly organized as *connected* and *partitioned*. Figure 7 shows a *heatmap* representing the OBUs density in the city for different periods of different days. As can be seen, both communication scenarios can occur. For instance, on Saturday at 8:00 we potentially have a connected scenario in the city center, while at 22:00 the OBUs are more spread out, characterizing a partitioned scenario. More detailed analysis of the OBU heat map can be found in the experiment evaluation section. Therefore, to analyze the multi-hop data dissemination algorithm and the infrastructure-based forwarding mechanism, we consider the following scenarios:

- Connected: the scenario in which it is possible to deliver the early warning messages using only V2V communications;
- Partitioned: the scenario in which it is not possible to deliver the early warning message promptly using the V2V communication;

To evaluate the network performance, we measure the following metrics:

- Delivery ratio: the percentage of warning messages successfully delivered to the target vehicles. It is desired that an efficient system delivers about 100% of its generated messages for managing traffic efficiently;
- Transmitted messages: the total number of messages transmitted by the system to guarantee its service. A high number of transmitted messages is an indication of redundant and unnecessary transmissions;
- Latency: time to deliver the warning message to a target vehicle. High latency degrades the system efficiency when dealing with traffic management.

For the end users who receive the early warning messages, they will adapt their routing decisions. The following metrics were used to analyze the efficiency of the system for improving end users’ travel experiences. In the calculation of the time loss, CO_2_ emission and fuel consumption, we used the model EMIT, integrated into the OMNeT++/SUMO simulation framework. The EMIT model integrated into SUMO was employed to calculate CO emissions and fuel consumption. The EMIT model is a simple statistical model for estimating the instantaneous emissions and fuel consumption of vehicles based on speed and acceleration and is derived from the Handbook Emission Factors for Road Transport (HBEFA) formula [43]. In general, they are all linearly dependent on the travel distances.

- **Travel time**: the total time that each OBU takes to travel a trajectory.
- **Time loss**: the total time spent in some traffic congestion and/or traffic bottleneck.
- **Travelled distance**: the total distance travelled by each OBU.
- **Fuel consumption**: the total fuel consumed by each OBU to travel its trajectory.
- **CO_2_ emissions**: the total CO_2_ emitted by each OBU to travel its trajectory.

## 5. Evaluation Results

In this section, we present and discuss the results of both the trajectory density predictor as well as the hybrid early warning system. To examine our density predictor, we mainly focus on prediction accuracy, whereas to evaluate the performance of the hybrid early warning system, we evaluate its network cost and traffic efficiency.

### 5.1. Trajectory Density Prediction Results

**Figure 8:**
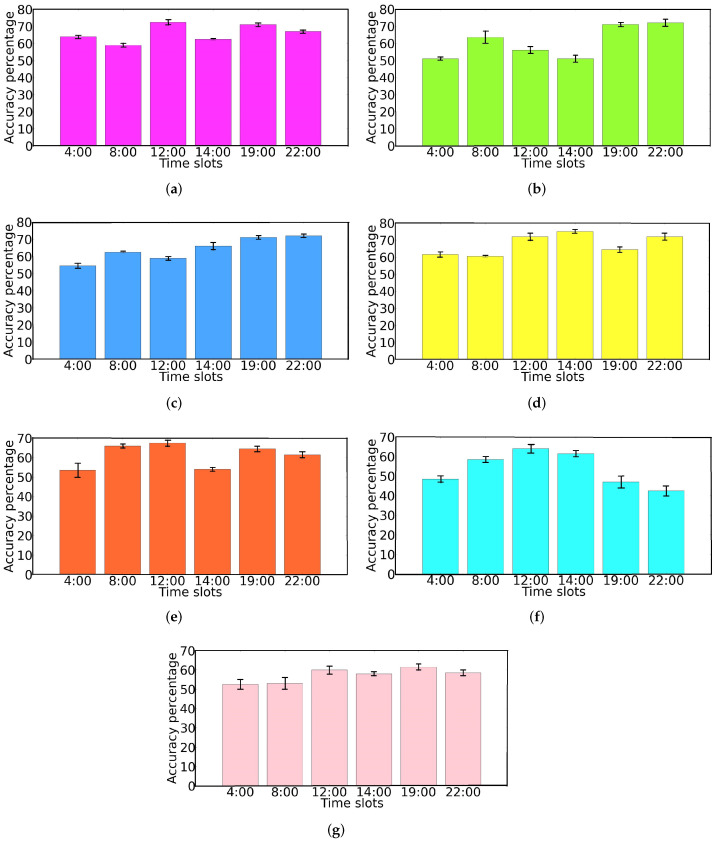
Trajectory density prediction accuracy of different days and time slots. (**a**) Monday; (**b**) Tuesday; (**c**) Wednesday; (**d**) Thursday; (**e**) Friday; (**f**) Saturday; (**g**) Sunday.

**Figure 9:**
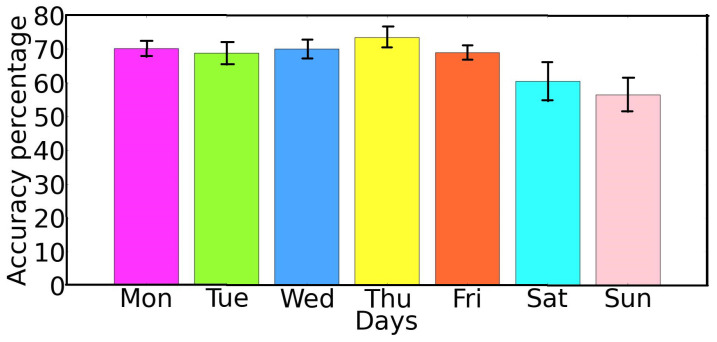
Average trajectory density prediction accuracy per each day.

To verify the trajectory density predictor, we conduct extensive experiments using a real-world VANET dataset. We use time granularity of 1 min ($\lambda_{density}=1$) to estimate the density of vehicles in trajectories for different time slots of each day. Figure 8 presents the accuracy of the proposed density predictor, for 100 OBUs on different days. As we can see, for most of the time slots on different days, our trajectory density predictor can deliver a satisfactory prediction accuracy of around 60% to 68% and the highest obtained prediction accuracy is around 75%. To clearly show the prediction performance of the proposed model, we calculate the average density prediction accuracy over different time slots for each single day. As we can see in Figure 9, for weekdays (*Monday to Thursday*), we reach an average prediction accuracy of 60% to 71%. However, for weekends (*Saturday and Sunday*), the prediction performance of the algorithm is slightly decreased (52% to 56%). This situation arises typically because during weekends road users and vehicles do not follow their daily movement patterns. Usually, they are taking different routes to travel among various locations, which shows the difficulty in exploring uncertain mobility patterns [35]. Therefore, our trajectory density predictor cannot make more accurate predictions on weekends. While during weekdays, road users move more regularly among frequently visited places, which makes it easier to generate accurate trajectory predictions.

In addition to obtaining the accuracy of predicted trajectory density for vehicles, we are also interested in identifying the number of OBUs that may take the same route to travel at a specific time slot of a day in the city of Porto. As shown in Figure 6, through experiments that we have conducted in the VANET dataset, we found that during weekdays vehicles are following almost the same traffic pattern. However, during weekends, traffic flows are different. Therefore, to predict the density of all OBUs, we randomly selected *Monday* and *Thursday* as weekdays, and *Saturday* as a weekend, and then we estimated the traffic pattern of all vehicles for different time slots per each selected day. As described before, Figure 7 presents the predicted density of vehicles for different time intervals of days. We discover that, in the early morning (4:00 a.m.), most vehicles are traveling in the *south-west* part of the city center. We observed that in the morning (8:00 a.m.) vehicles tend to take most of the trajectories that are passing through the city center. Besides, the predicted density area is more extensive than the area that is detected at 4:00 a.m. According to our predictions, during mid-day (12:00 p.m.) most vehicles are still traveling along the routes that are passing through the city center. However, in the evening (10:00 p.m.), most vehicles’ flows are scattered among the touristic center (*west side of the city*) and the city center.

### 5.2. Network Performance Results

**Figure 10:**
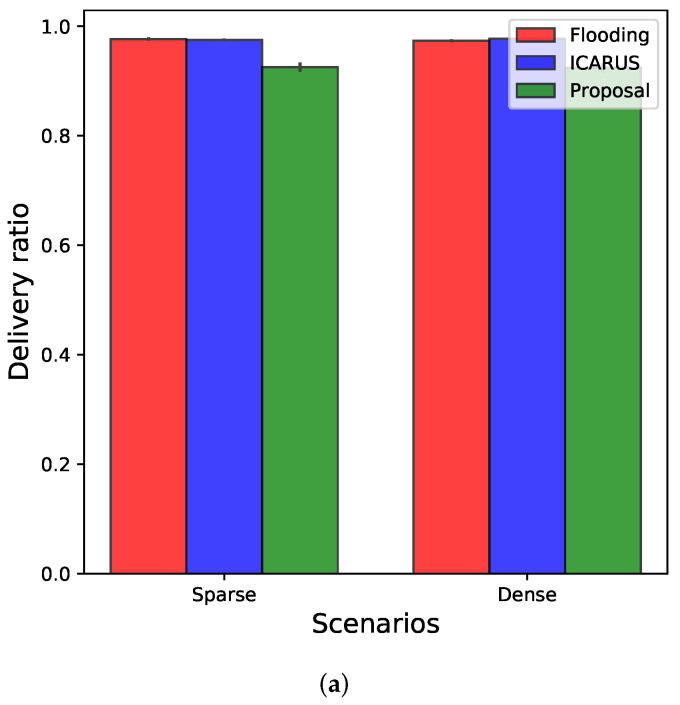

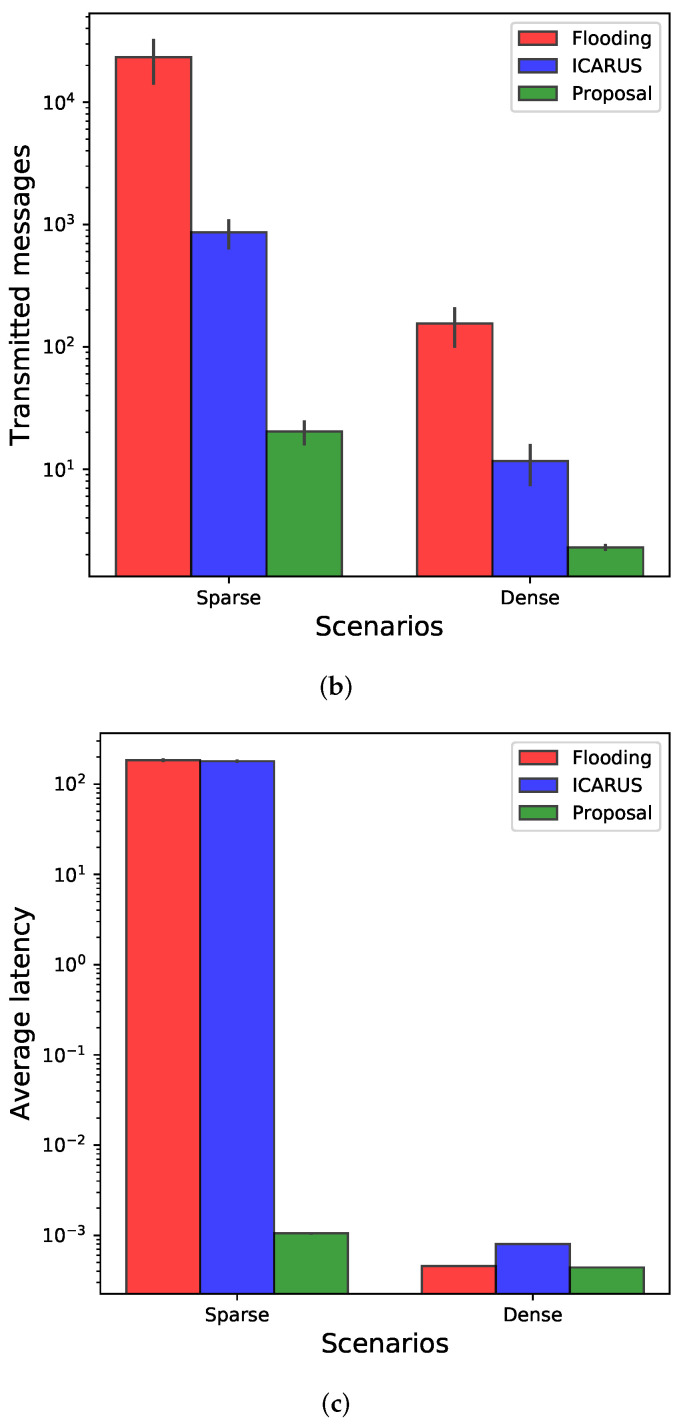
Network performance results for both sparse and dense scenarios. (**a**) Delivery ratio; (**b**) Transmitted messages; (**c**) Latency.

To evaluate the network performance of the proposed hybrid early warning system we compared its results with Flooding and ICARUS [20]. Figure 10 shows the average results considering all assessed metrics in both scenarios partitioned and connected. As can be seen, all solutions present a delivery ratio higher than 90%. However, as our proposal considers the density predictions to perform the data dissemination, in the case of mis-predictions it potentially cannot deliver the warning message accurately, which will decrease the delivery ratio by approximately 5% when compared to Flooding and ICARUS in both scenarios (as shown in Figure 10a). This is because when mis-prediction happens, the warning message will be disseminated to wrong locations, while the delay of this mis-dissemination will not be affected, as far as the network connectivity of V2V or V2I is not changed. Therefore, we observe that the early warning message is not delayed, but rather be delivered to the wrong locations, which leads to the reduced delivery ratio of 5%.

Yet, we can see the efficiency of the proposed system when analyzing the transmitted messages (Figure 10b) and the latency (Figure 10c) for delivering the traffic incident messages. As expected, Flooding has the highest number of transmissions because all OBUs that receive the warning message rebroadcast it, consequently producing a high number of redundant and unnecessary transmissions. Therefore, in partitioned scenarios, in which Flooding needs to replicate the transmission of the incident message periodically to ensure the delivery to all relevant vehicles (e.g., the set of OBUs predicted to pass the incident location), the number of transmissions dramatically increases.

On the other hand, considering latency results, Flooding presents low latency in connected scenarios because it does not introduce any additional delay in the multi-hop data forwarding, thus the vehicles rebroadcast the incident message as soon as they receive it. However, in partitioned scenarios, an undesired latency is introduced; since the multi-hop dissemination is not possible, the source OBU (e.g., the OBU involved in the unexpected traffic incident) needs to wait for the relevant vehicles to be within its coverage to forward the warning message. In particular, in connected scenarios, Flooding presents an average latency of 300 milliseconds, while in partitioned scenarios Flooding presents an average latency of approximately 200 s.

Unlike Flooding, ICARUS employs an efficient broadcast suppression mechanism to avoid that all OBUs that receive the warning rebroadcast it. Hence, ICARUS reduces the number of transmissions by 70% when compared to Flooding. However, as ICARUS only relies on V2V communications to deliver the warning messages, in partitioned scenarios the source OBU also needs to rebroadcast the warning periodically, consequently increasing the number of transmissions (see Figure 10b). Therefore, the latency results for partitioned scenarios in ICARUS potentially has the same behavior as Flooding (e.g., it increases the latency because the multi-hop dissemination is not possible). However, for connected scenarios, ICARUS presents a higher latency than Flooding because it introduces a small delay in each hop to enable the broadcast suppression (see Figure 10c). This delay is based on the distance between the transmitting and receiving OBU, allowing the OBUs that received the warning message to wait for a few milliseconds to know whether or not the rebroadcast of the incident message is necessary.

Finally, different from ICARUS and Flooding, our proposal can use the multi-hop and infrastructure-based forwarding. Hence, it can decrease the number of transmissions and the latency even in partitioned scenarios. Besides, due to the efficient data dissemination algorithm based on vehicles’ trajectories and traffic density predictions, our proposal selects the most efficient set of relay OBUs that will promptly rebroadcast it as soon as they receive the incident message, consequently providing a low latency in connected scenarios. On the other hand, in partitioned scenarios, the system forwards the incident message to the next RSU that the relevant vehicle will pass, decreasing the latency. Moreover, the proposed data dissemination algorithm only sends the warning message towards the relevant vehicles’ direction (since the system knows where each relevant vehicle is located based on predictions), thus reducing the number of necessary transmissions to deliver the warning accurately. However, Flooding and ICARUS need to disseminate the message in all directions to find the relevant vehicles, increasing the number of transmissions. In particular, our proposal can reduce the number of transmissions and the latency by 95% and 98% when compared to Flooding and ICARUS, respectively, in partitioned scenarios.

### 5.3. Traffic Efficiency Results

**Figure 11:**
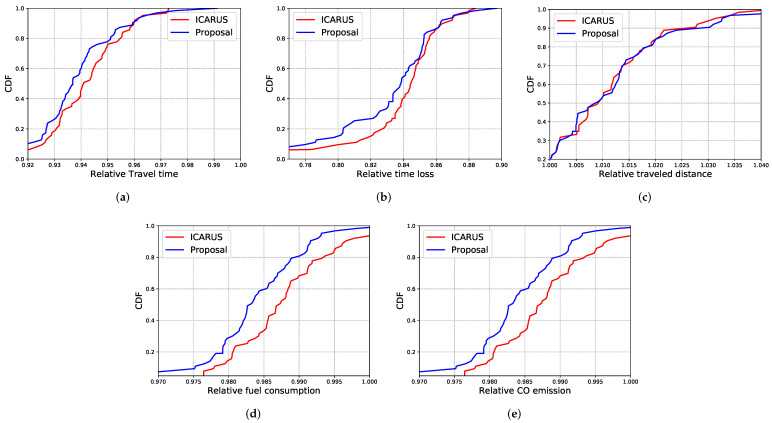
Traffic efficiency results. (**a**) Travel time; (**b**) Time loss; (**c**) Traveled distance; (**d**) Fuel consumption; (**e**) CO emission.

To evaluate the traffic efficiency of the proposed system, we have compared its results with ICARUS [20]. Figure 11 shows the results of the assessed metrics as relative values (e.g., the ratio between the mobility with a solution to avoid the unexpected traffic incident and the mobility of real mobility extracted from the dataset) in a Cumulative Distribution Function (CDF).

As expected, both ICARUS and the proposed system present better results for all assessed metrics when compared to the real mobility extracted from the dataset. This is a result of the knowledge about the unexpected traffic incident delivered to the vehicles and also to the re-routing mechanism employed by them to avoid the incident area. Therefore, the vehicles will not become stuck in the congestion produced by the traffic incident, consequently decreasing their travel time as well as time loss (see values smaller than 1 in Figure 11a,b). To avoid the incident location, the vehicles increase their traveled distance by approximately 5% (see Figure 11c values greater than 1). However, such increasing in the traveled distance is not an issue, since it not only improves the overall traffic efficiency, but also decreases fuel consumption and CO_2_ emissions (see Figure 11d,e).

The slight improvement in the assessed metrics achieved by the proposed system in comparison to ICARUS is because our system can deliver the warning messages earlier than ICARUS even in partitioned scenarios, consequently enabling the relevant vehicles to change their route and improve their mobility within an appropriate amount of time. However, due to the limited performance of ICARUS in partitioned scenarios, in some cases, the vehicles can be informed too late about the traffic incident, not allowing them to perform a detour to avoid the traffic incident location. In this context, due to the lower overhead produced by the proposed system and to its traffic efficiency, we can conclude that knowing in advance the vehicle’s trajectory and the road density can provide substantial improvements for ITS applications, not only for improving traffic efficiency, but also for improving network performance.

## 6. Conclusions

This work proposes an effective safety message dissemination system by using machine learning-empowered vehicle trajectory density prediction information. With the proposed system, the traffic safety alarming message will only be disseminated to relevant vehicles that are predicted to pass through the accident areas. Depending on the network connectivity, the system adaptively chooses either the V2V or V2I approach to disseminate the message to relevant vehicles to ensure timely message delivery. We evaluate the system by using a real-world VANET mobility dataset. Simulation results showed that our system outperforms other dissemination mechanisms without considering the predicted vehicle trajectory density information.

For future work, we will investigate the following two aspects: (1) model the trajectories of urban traffic as a graph and define a traffic graph convolution operation to capture spatial and temporal features from the traffic network; and (2) further improvements of the message dissemination mechanism together with the predicted trajectories’ traffic flow.

## Figures and Tables

**Table 1:** Simulation parameters.

Parameters	Values
OSM bounding box	41.1790, −8.6912; 41.1390, −8.5765
Channel frequency	5.89e0 Hz mW
Propagation model	Two ray
Transmission power	2.2 mW
Communication range	300 m
Bit rate	18 Mbit/s
PHY model	IEEE 801.11p
MAC model	EDCA

## Data Availability

Not applicable.
